# Sacrococcygeal Teratoma

**Published:** 2013-04-01

**Authors:** V Raveenthiran

**Affiliations:** Department of Pediatric Surgery, SRM Medical College and Hospital SRM University, Chennai, India.

 (Athena stands for abbreviation of Abstracting and Thoughtful Evaluation of Neonatal Articles; but it is also personified by the contributor. Like Athena of Greek mythology, she distills wisdom from published literature)

Athena is fascinated by the tailbone (coccyx) for more than one reason: it is named poetically after the beak of a cuckoo; it is the key Darwinian link between the tail of primates and the tailless Homo sapiens; notwithstanding its tiny size it plays a vital role in anal continence by giving attachment to anal sphincters; and above all it harbors totipotent stem cells giving rise to a peculiar tumor - the Sacrococcygeal Teratoma (SCT). This tumor is peculiar because it is almost always benign to start with and it turns malignant with passage of time. This emphasizes the importance of early surgery and the need to excise coccyx in SCT. Athena believes that studying the natural history of SCT may unravel the secrets of oncogenesis. Her curiosity is perpetuated by recent reports [1 - 16] describing as to how these tumors have been mistaken for a variety of other lesions and vice versa (Table 1).

**Figure:**
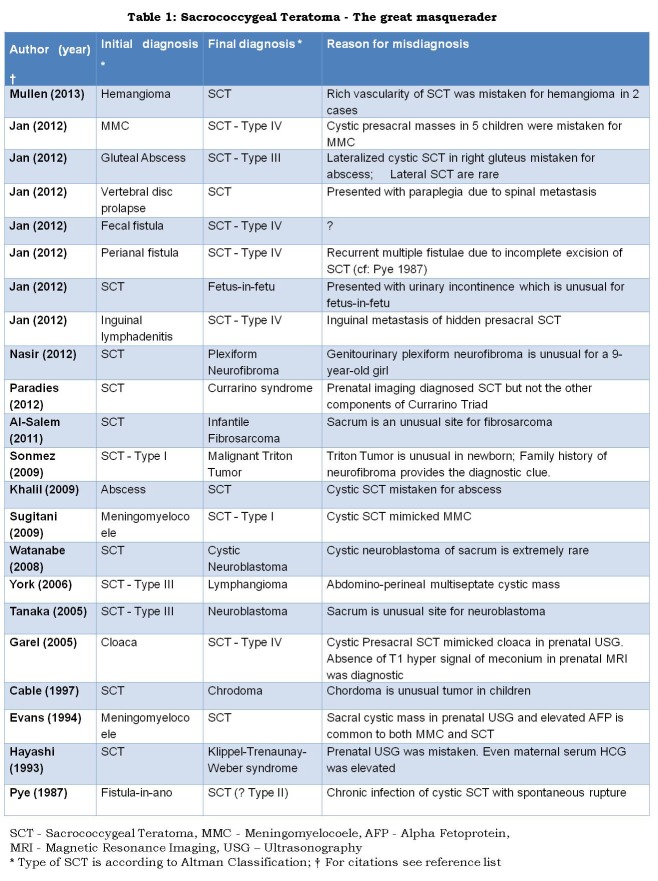
Table 1: Sacrococcygeal Teratoma - The great masquerader

Recently, Shalaby et al [17] reported a mysterious vanishing of mid-urethra in girls undergoing excision of SCT. In a series of 53 SCT collected over a period of 30 years, they described 5 girls who had “vanishing mid-urethra”. In all of them the proximal urethra had fistulous communication with vagina. In 4 of them the external urethral meatus was normal and the distal urethra was ending blindly; one had complete absence of the distal urethra. Interestingly, in none of them the anomaly was recognized preoperatively despite the fact that all of them had been catheterized in the perioperative period. The diagnostic delay ranged from 6 weeks to 13 years after SCT excision. All of them presented with voiding dysfunction (2 with retention of urine and 3 with incontinence). Importantly, the diagnosis was elusive even after initial cystoscopic examinations. This phenomenon has also been previously reported in 2 girls [18]. 

Hypothetical explanations of this phenomenon include: (i) congenital malformation of the urogenital sinus due to the mechanical presence of teratoma in the fetal pelvis, (ii) iatrogenic injury to urethra and (iii) pressure necrosis of the mid-urethra that is compressed between pubic symphysis and the tumor. Intactness of proximal and distal parts of the urethra rules out the possibility of interference with urethral embryogenesis. Absence of concomitant injury to rectum and absence of intraoperative difficulties as recorded by experienced surgeons makes iatrogenic injury unlikely. If pressure necrosis is the cause, then why is it not occurring in boys? Therefore, Athena surmises that it could be due to unrecognized iatrogenic intraoperative injury to specific branches of the middle rectal artery. Branches of this artery supplies middle one third of vagina and the adjoining mid-urethra in females. Athena’s guess is supported by the fact that one of the patients in Shalaby’s series developed concomitant rectosigmoid stricture and mid-vaginal stenosis following SCT excision. The proximal urethra is usually supplied by the inferior vesical artery and the distal urethra by internal pudendal artery. Intactness of these arteries perhaps explains as to why the proximal and distal one third of the urethra is preserved in “Vanishing mid-urethra syndrome”. Rich collaterals between inferior vesical artery and bulbar artery probably protect the male urethra even if the middle rectal artery is damaged during surgical excision of SCT. 

SCT is fraught with several serious complications. Huge tumor distorting the anatomy, extensive pelvic dissections and tumor infiltration or encasement of pelvic nerves leads to numerous complications such as bowel and bladder dysfunction, sexual inadequacy, gait alteration, distorted gluteal mound, wound dehiscence and death due to excessive intraoperative blood loss or malignant recurrences. In the last decade (2003 - 2013) several authors [19 - 35] reported their long-term results with SCT excision (Table 2). Mean follow-up in some of the series was as long as 25 years. But these impressive studies are far from bringing the good news. Although mortality is less than 10% in most of the reports, continence and cosmesis are of great concern. Approximately one third of patients suffer long-term incontinence of stool and urine. In some of the studies [22, 25] fecal and urinary incontinence is as high as 64% and 50% respectively. Athena wonders as to what proportion of post-surgical female urinary incontinence can be attributed to missed diagnosis of “vanishing mid-urethra”.

**Figure:**
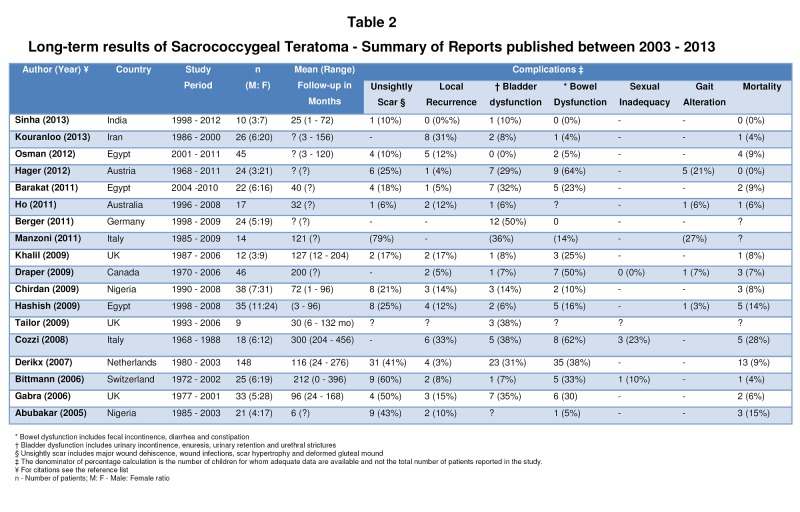
Table 2: Long-term results of Sacrococcygeal Teratoma - Summary of Reports published between 2003 - 2013

One study from Ghana [36] reports a wound dehiscence rate as high as 90%. To Athena’s dismay most of the studies do not even mention sexual inadequacy and gait related problems. Bittmann et al [33] recorded obstetric difficulty due to rigid pelvic-outlet in one of their patients (10%). Zaccara et al [37] did gait analysis of SCT patients using Vicon 3-D motion analysis system. Although children exhibited an apparently normal gait following SCT excision, kinesiometric analysis showed significant reduction in hip extension, range of ankle movements and knee power. Paradoxical increase in ankle power was also noticed. Precise definitions and rigorous postoperative evaluation are missing in all most all the studies. Athena fears that a more stringent follow-up protocol may increase the complication rates in excess of 50%. 

Approximately 3 to 31% of SCT have a tendency to recur after excision. Derikx et al [38] and De Backer et al [39] studied the factors responsible for tumor recurrence in a cohort of 173 and 70 children respectively. Incomplete excision [38], failure to excise coccyx [39] and malignant or immature histology have emerged as risk factors of tumor recurrence [38, 39]. Microscopic residue of mature or immature teratoma at the resection margin was rarely associated with local recurrences; however, presence of yolk sac tumor (YST) component was an exception to this rule [39]. In case of cystic teratomas spillage of solid components rather than the cyst fluid was responsible for recurrences [39]. According to Derikx et al [38] tumor size, Altman type and age at diagnosis are not associated with recurrence. In this context, Athena is reminded of a previous paper wherein Bilik et al [40] have showed that a large tumor may possibly have imperceptibly tiny foci of malignant cells; missing these tiny foci during histological sectioning may erroneously label the tumor as benign. In huge tumors it is impractical to histologically sample and study every millimeter of the lesion. In the background of this contention Athena deems it wise to consider large tumor size as a risk factor of local recurrence even though Derikx’s data do not support that notion. Is it not true that wisdom is beyond the realms of statistics and evidences? 

Early detection of recurrence is often facilitated by serial estimation of tumor markers during follow-up. The role of alpha fetoprotein (AFP) and human chorionic gonadotropin (HCG) in the follow-up of SCT is well known. Pauniaho et al [41] studied the role of various tumor markers in a cohort of 32 children with 1 to 15 years of follow-up. Six of the children had 8 recurrences. AFP was useful in detecting malignant recurrences while CA-125 was useful in early detection of recurrences of mature and immature tumors. Athena would recommend complementing AFP with CA-125 in post-surgical follow-up of SCT. 

Rich vascularity and huge tumor size often cause fetal circulatory failure and hydrops. Recently several parameters, based on prenatal sonography, have been developed to prognosticate the fetal outcome in SCT. Sy et al [42] proposed a ratio between the volume of the tumor and the fetal head. In case of cystic SCT, only the volume of solid component was taken into consideration. There were no deaths when the tumor-head ratio (THR) was less than 1; alarmingly the mortality was 61% when the THR was more than 1. Rodriguez et al [43] suggested a ratio of tumor volume and fetal weight. The tumor-fetal ratio (TFR) was predictive of fetal outcome before 24 weeks of gestation. TFR < 0.12 had a significantly better outcome than TFR > 0.12. The sensitivity and specificity of this prediction were 100% and 83% respectively. Despite the limitation of small sample size in these studies, Athena believes these parameters be of use in offering evidence based prenatal counseling.

## Footnotes

**Source of Support:** Nil

**Conflict of Interest:** The author is Editor of the journal. But he did not take part in the evaluation or decision making of this manuscript. The manuscript has been independently handled by two other editors.
